# Investigation on Cutting Fluid Penetration Kinetics and Friction Reduction Mechanism of Micro-Textured Tools via Direct Vapor-Phase Capillary Filling

**DOI:** 10.3390/ma19173694

**Published:** 2026-08-30

**Authors:** Dongliang Ge, Jiankang Ma, Aihua Liu, Zhengyi Tang, Jiaxing Wu, Yanqiang Sun, Yuhao Zhang

**Affiliations:** 1 School of Construction Machinery, Shandong Jiaotong University, Jinan 250357, China; 2 Shandong Key Laboratory of Technologies and Systems for Intelligent Construction Equipment, Shandong Jiaotong University, Jinan 250357, China

**Keywords:** micro-textured tool, tool–chip interface, capillary penetration, vapor-phase filling, friction reduction

## Abstract

Severe friction and extreme temperatures occur at the tool–chip interface during metal cutting. Conventional cutting fluids struggle to penetrate interface micro-capillaries at the contact interface under high contact pressure. This issue causes severe tool–chip adhesion and accelerates tool wear. This study aims to solve fluid delivery limitations by introducing micro-textures with a depth of 15 microns on the tool surfaces. It reveals the mechanism of micro-textures in accelerating fluid penetration and reducing interface friction. An analytical capillary penetration model was established for conventional and micro-textured tools. Thermal penetration tests (30–150 °C) and turning experiments on hardened steel were conducted to evaluate interfacial fluid transport behavior. Theoretical modeling shows that micro-textures facilitate direct vapor-phase filling into micro-capillaries. This mechanism bypasses liquid ingress and droplet evaporation stages. This reduces the fluid penetration time into the capillaries by almost an order of magnitude. Thermal tests show that textured surfaces maintain dynamic vapor–liquid equilibrium. At 150 °C, the vapor penetration area reaches 978.5 × 10^−3^ mm^2^ on micro-textures, over four times that of smooth surfaces. Energy dispersive spectrometry (EDS) detected fluid-derived sodium (0.98 at.%) inside micro-textured capillaries. Meanwhile, workpiece material adhesion decreases from 6.04 at.% to 0.11 at.%. In turning tests of AISI 1045 hardened carbon steel, micro-textured tools reduced the main cutting force by up to 17% and the axial force by up to 22%. Cutting temperatures decreased by up to 10.0%. The average tool–chip friction coefficient dropped by 9.2% at a cutting speed of 240 m/min. This work provides insights into a vapor-phase lubrication mechanism and offers quantitative guidance for designing high-efficiency self-lubricating tools.

## 1. Introduction

Metal cutting generates extreme thermal and mechanical stresses at the tool–chip interface [1,2]. High contact pressures and elevated temperatures cause severe material adhesion and rapid tool wear [3,4]. These severe conditions degrade workpiece surface quality and shorten tool lifespan [5]. Cutting fluids are widely used to cool the cutting zone and reduce friction [6]. They form a protective boundary lubrication film at the partial contact interface [7]. However, extreme compressive pressures prevent liquid cutting fluid from entering the inner contact zone [8,9]. Dry sliding friction often dominates the tool–chip interface. Consequently, conventional lubrication with cutting fluid cannot provide effective lubrication [10]. Enhancing fluid penetration into the interface micro-capillaries is essential to reduce friction and tool wear.

Researchers have explored various strategies to improve cutting fluid penetration [11,12,13,14,15,16]. On the one hand, advanced delivery methods have been developed, including minimum quantity lubrication (MQL), high-pressure cooling, and nanofluids. These methods increase fluid momentum and thermal capacity. However, they do not change the intimate contact mechanics at the tool–chip interface. On the other hand, tool surface micro-texturing technologies have been widely applied [17,18]. Micro-grooves and micro-dimples were fabricated on tool surfaces using laser or electrical discharge machining [19]. These micro-textures reduce the real contact area, trap wear debris, and act as fluid reservoirs [20]. Nevertheless, under severe cutting conditions, plasticized workpiece material often clogs these micro-structures, degrading their friction-reduction performance over time [21]. Furthermore, classical capillary penetration models have been established to understand fluid transport [22]. Williams [1] proposed an interconnected rectangular capillary network model for interfacial fluid flow. Godlevski et al. [2] developed a single cylindrical capillary model. This model divides fluid transport into three sequential stages: liquid phase ingress, micro-droplet evaporation, and vapor phase filling. These fundamental models confirm that capillary penetration kinetics determine boundary lubrication efficiency [22].

Despite these advances, a critical research gap remains [23]. The coupling mechanism between surface micro-textures and fluid penetration kinetics under extreme gradients is not fully understood [24]. Classical models assume that fluid transport must follow a sequential path [2,25]. The fluid must first undergo liquid penetration and then micro-droplet evaporation [2,26]. This multi-stage process requires significant time under high contact pressure [27]. It remains unclear how surface micro-textures alter fluid phase transitions near the contact zone [28]. Specifically, the mechanism by which micro-textures shorten capillary transport time and lower boundary shear stress needs further explanation. Direct quantitative evidence of vapor-phase transport in micro-textured tool capillaries is still scarce.

To address these issues, this study investigates the fluid penetration kinetics and friction reduction mechanism of micro-textured Al_2_O_3_/TiC ceramic tools. An analytical capillary penetration model is first established for conventional and micro-textured tools. The model introduces a direct vapor-phase filling mechanism. This mechanism bypasses the liquid ingress and micro-droplet evaporation stages, significantly shortening penetration time. Next, static thermal penetration tests (30–150 °C) are conducted using an optical visualization system. These tests evaluate temperature-dependent fluid spreading and phase distributions on textured surfaces. Finally, hard turning experiments on hardened steel (*v* = 40–240 m/min) are performed. Cutting forces, cutting temperatures, and friction coefficients are measured. Energy dispersive spectrometry (EDS) is used to detect fluid-derived sodium (Na) inside interface micro-capillaries and evaluate adhesion mitigation [29]. This work provides experimental and theoretical insights consistent with accelerated vapor-phase transport on micro-textured tools. It offers a quantitative foundation for designing high-efficiency self-lubricating tools.

## 2. Theoretical Framework and Analytical Modeling

### 2.1. Tool–Chip Interface Contact Mechanics and Stress Distribution

**Figure 1:**
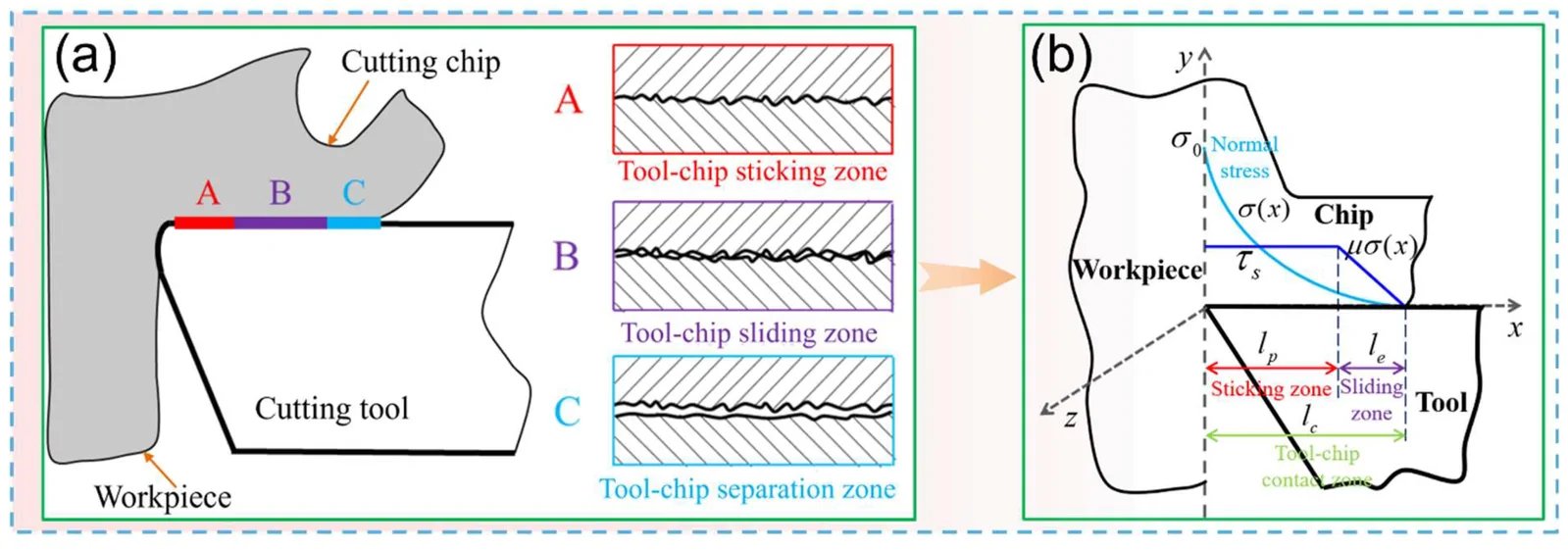
Interfacial contact mechanics and stress distribution models at the tool–chip interface: (**a**) sticking (*l_p_*) and sliding (*l_e_*) contact zone definitions; (**b**) normal stress *σ*(*x*) and shear stress τ(x) distribution curves along the tool rake face.

Establishing a reliable tool–chip contact model is essential to analyze interfacial friction [21]. Figure 1 illustrates the interfacial contact mechanics, section definitions, and stress distribution models at the tool–chip contact interface. Under severe cutting conditions, high contact pressure and elevated temperatures cause material adhesion and abrasive wear near the cutting edge [22]. The tool–chip contact zone of length *l_c_* is divided into a sticking zone (length *l_p_*) and a sliding zone (length *l_e_* = *l_c_* − *l_p_*) [21,22]. According to the modified Coulomb friction model, the shear stress distribution *τ*(*x*) along the rake face is expressed as

(1) $$\tau (x)=\left\{\begin{array}{c} \;\;\;\tau_{s},\;\;\;\;\;\;\;0\leq x\leq l_{p}\;\;\;\;\;\;\;(\mathrm{sticking}\;\mathrm{zone})\\ \mu \sigma (x),\;\;\;l_{p}<x\leq l_{c}\;\;\;\;\;\;(\mathrm{sliding}\;\mathrm{zone}) \end{array}\right.$$

where *τ_s_* is the shear yield strength of the workpiece material, *μ* is the friction coefficient in the sliding zone, and *x* is the distance from the cutting edge [21]. The normal stress distribution *σ*(*x*) along the rake face follows a power-law relationship:

(2) $$\sigma (x)=\sigma_{0}{\left(1-\frac{x}{l_{c}}\right)}^{\xi }$$

where *σ*_0_ is the peak normal stress at the tool edge, and *ξ* is the stress distribution exponent (*ξ* = 2 or 3) [22].

### 2.2. Cutting Force and Interfacial Temperature Mechanics

**Figure 2:**
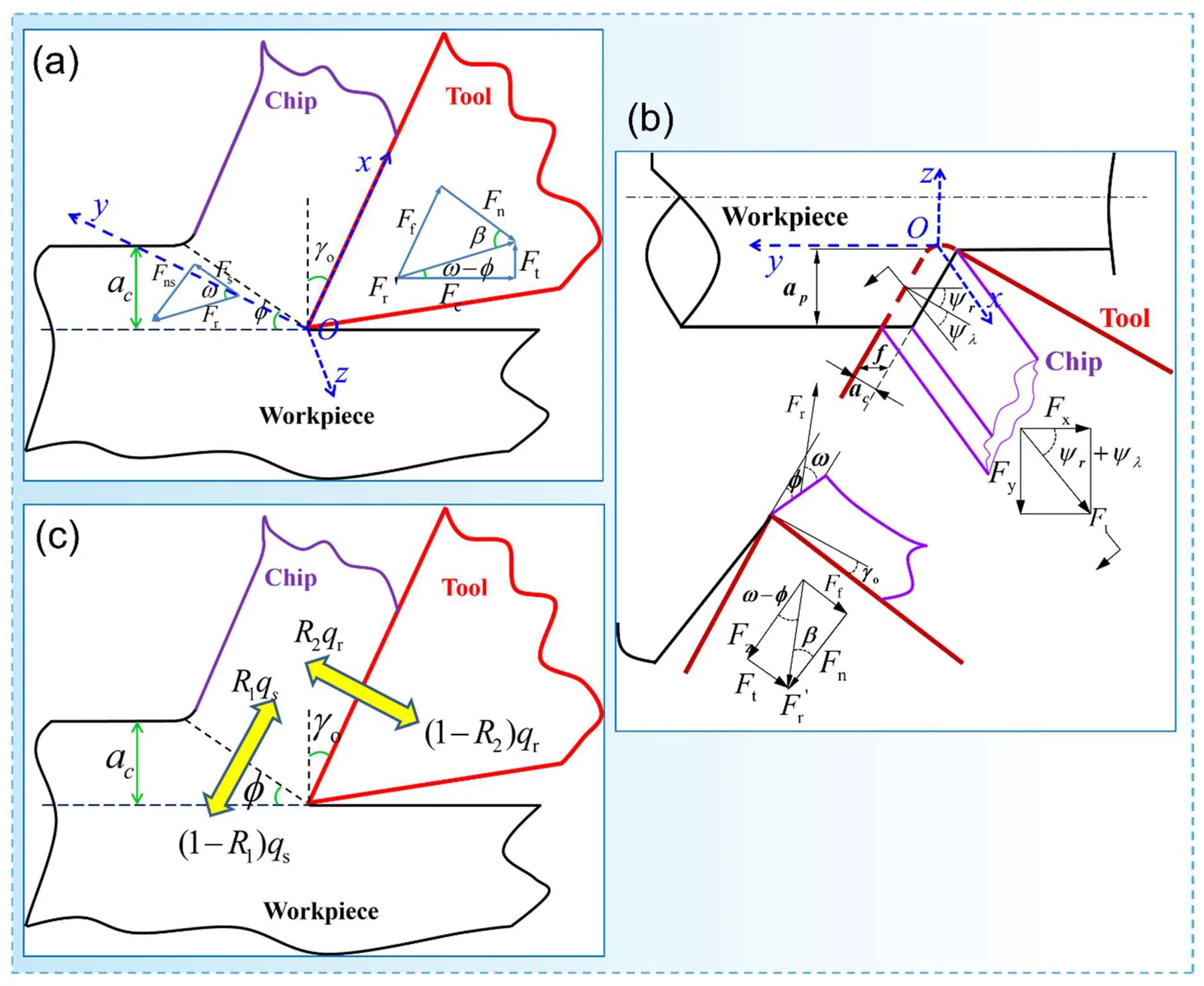
Cutting force mechanics and heat flux partitioning in metal cutting: (**a**) force balance in orthogonal cutting; (**b**) force resolution model in oblique turning; (**c**) heat generation and heat flux partition scheme between workpiece, tool, and chip.

Figure 2 details the cutting force resolution and thermal flux partitioning schemes during metal cutting. In metal cutting, cutting forces and temperatures are directly governed by the average shear stress ${\bar{\tau }}_{c}$ and the friction force *F_f_* at the tool–chip interface [23]. Based on orthogonal cutting force equilibrium, the total friction force *F_f_* acting on the rake face is given by

(3) $$F_{f}={\bar{\tau }}_{c}\cdot A={\bar{\tau }}_{c}\cdot l_{c}\cdot \alpha_{w}$$

where *A* is the total tool–chip contact area, *l_c_* is the total contact length, and *α_w_* is the cutting width [23]. For oblique turning processes, three-component cutting forces—axial force *F_x_*, radial force *F_y_*, and main cutting force *F_z_*—are derived from the resultant force $F_{r}^{\prime }$ and tool geometry angles [24]:

(4) $$F_{x}=F_{r}^{\prime }\cos(\omega -\phi )\sin\psi_{r}+F_{f}\cos\psi_{\lambda }$$

(5) $$F_{y}=F_{r}^{\prime }\cos(\omega -\phi )\cos\psi_{r}-F_{f}\sin\psi_{\lambda }$$

(6) $$F_{z}=F_{r}^{\prime }\sin(\omega -\phi )$$

where *ψ_r_* is the tool cutting edge angle, *ψ_λ_* is the chip flow angle, *ϕ* is the shear angle, and *ω* is the angle between the resultant force and the shear plane [24].

The average cutting temperature ${\bar{\theta }}_{t}$ in the cutting zone arises from plastic deformation energy in the primary shear zone and frictional work along the rake face [24]:

(7) $${\bar{\theta }}_{t}=\theta_{0}+{\bar{\theta }}_{s}+{\bar{\theta }}_{r}$$

where ${\bar{\theta }}_{0}$ is the ambient temperature, ${\bar{\theta }}_{s}$ is the average temperature rise caused by shear deformation, and ${\bar{\theta }}_{r}$ is the frictional temperature rise at the tool–chip interface. The frictional heat generation rate *q_r_* is directly proportional to the friction force *F_f_* and the chip sliding velocity *v_c_*.

### 2.3. Penetration Kinetics in Conventional Tool Capillaries

**Figure 3:**
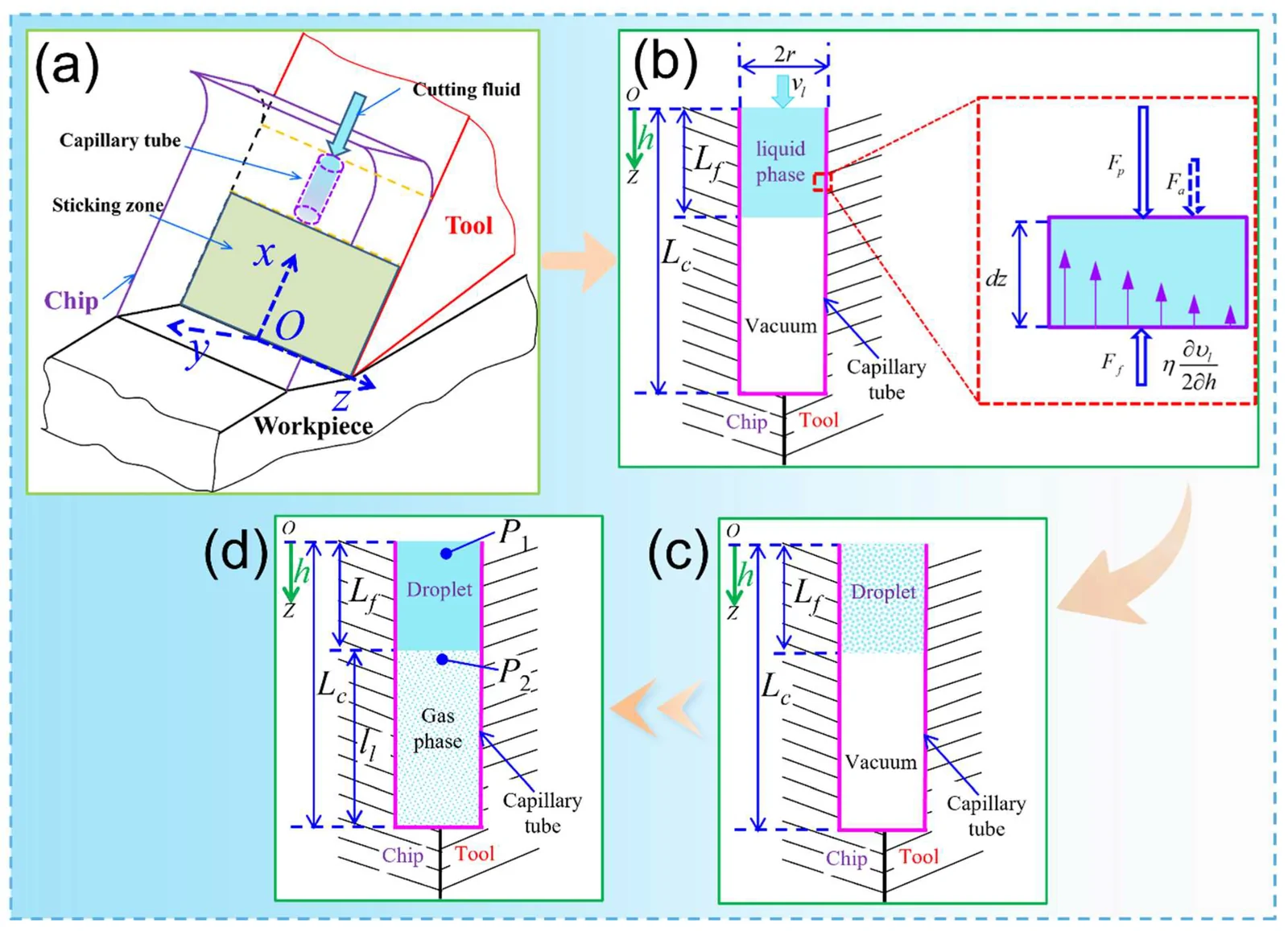
Multi-stage penetration kinetics of cutting fluid in conventional tool capillaries: (**a**) single cylindrical capillary model; (**b**) Stage I liquid phase ingress under external pressure; (**c**) Stage II micro-droplet vaporization; (**d**) Stage III vapor transport.

Figure 3 presents the multi-stage penetration kinetics of cutting fluids inside conventional tool micro-capillaries. Interfacial asperities between the rake face and chip form a network of micro-capillaries [2,25]. In conventional untextured tools, cutting fluid penetrates these micro-capillaries through a sequential three-stage process.

**Stage I: Liquid Ingress.** Under applied pressure *P_z_*, the liquid phase enters a single cylindrical capillary of radius *r* [25]. Force balance on a liquid element yields:

(8) $$F_{p}-F_{f}=F_{a}\Rightarrow \pi r^{2}dP_{z}-2\pi rdz\cdot \eta \frac{\partial \upsilon_{l}}{\partial h}=\rho_{l}\pi r^{2}dz\frac{\partial \upsilon_{l}}{\partial t}$$

where *F_p_* is the external pressure force, *F_f_* is the internal viscous friction force, *F_a_* is the acceleration force, *η* is dynamic viscosity, *ρ_l_* is fluid density, and *v_l_* is liquid penetration velocity. Dimensional analysis yields the liquid penetration time *τ_l_*:

(9) $$\tau_{l}=K\cdot \frac{a\cdot h}{\nu }\cdot Pr^{n}$$

where *K* is a dimensionless constant, *a* and *h* are capillary geometry parameters, *v* is kinematic viscosity, and *P_r_* is the Prandtl number.

**Stage II: Micro-Droplet Evaporation.** Upon reaching high-temperature zones, liquid micro-droplets undergo intense vaporization. Energy conservation gives the initial vapor velocity *υ_g_*_0_:

(10) $$\upsilon_{g0}=\sqrt{2T(C_{pm}-C_{Vpm})}$$

where *T* is the local absolute temperature in Kelvin (K), *C_pm_* is specific heat at constant pressure, and *C_Vpm_* is specific heat at constant volume [26].

**Stage III: Vapor Transport.** The generated vapor fills the gas-filled capillary length *l_l_*. Based on the ideal gas law and viscous gas flow momentum equations, the vapor filling time *τ_g_* is derived as

(11) $$\tau_{g}=\frac{\mu_{g}\cdot l_{l}^{2}}{r^{2}(P_{atm}+\Delta P)}\cdot \ln(\frac{P_{1}}{P_{2}})$$

where *μ_g_* is gas viscosity, *l_l_* is the gas-filled capillary length, *P_atm_* is atmospheric pressure, and Δ*P* is the pressure differential; *P*_1_(*P*_1_ = *P_atm_* + Δ*P*) is the entrance pressure, and *P*_2_ is the vapor front pressure. The total penetration time for conventional tools is the sum of all three stages: $\tau_{total}=\tau_{l}+\tau_{evap}+\tau_{g}$ [2,26].

### 2.4. Direct Vapor-Phase Filling Mechanism in Micro-Textured Tools

**Figure 4:**
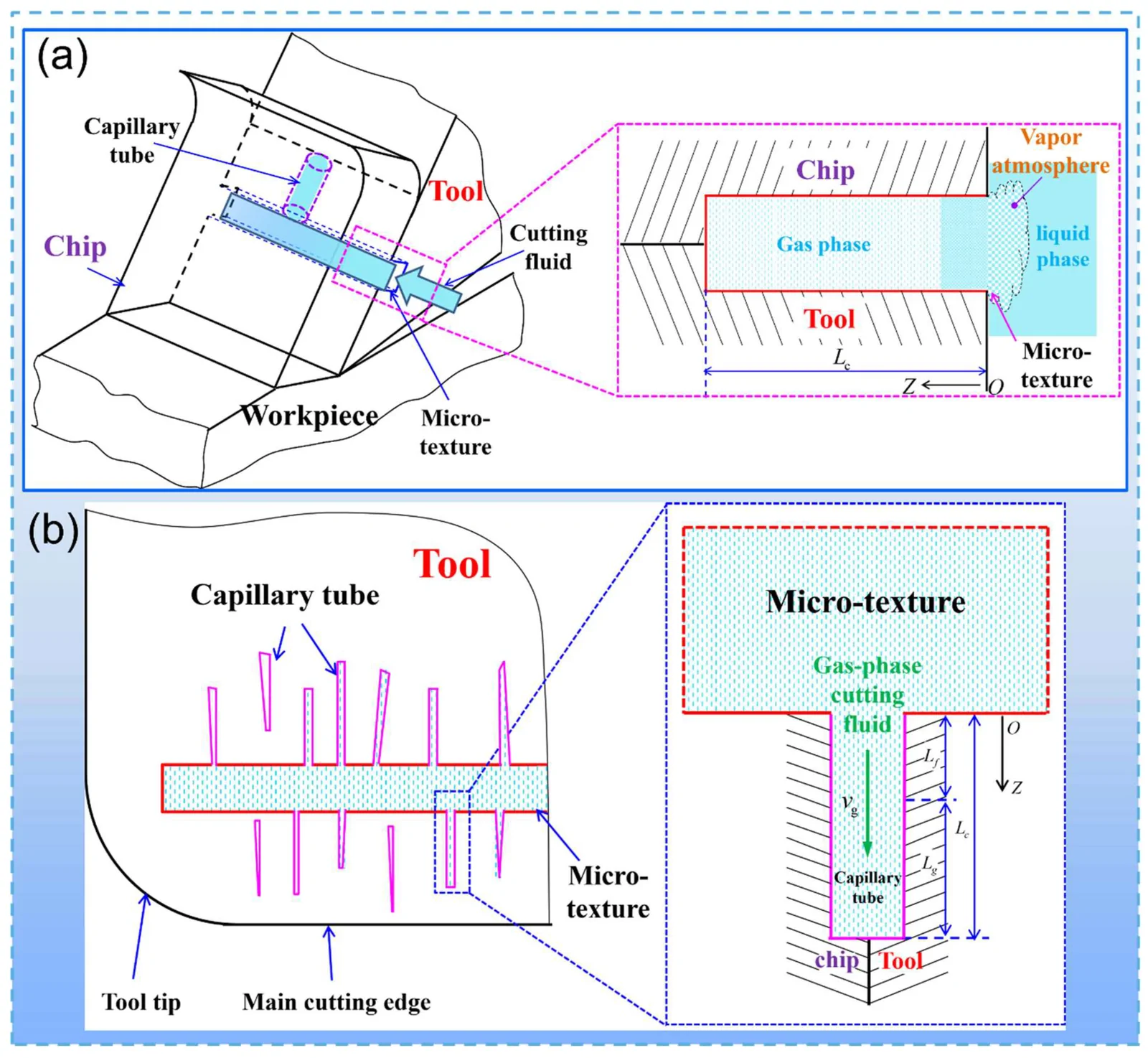
Direct vapor-phase filling kinetics and friction reduction mechanisms in micro-textured tools: (**a**) formation of localized vapor cushion inside micro-grooves; (**b**) direct vapor-phase filling into adjacent contact capillaries.

Figure 4 depicts the direct vapor-phase filling kinetics and friction reduction mechanisms in micro-textured tools. Surface micro-textures fundamentally alter the fluid transport pathway. Micro-grooves serve as localized fluid reservoirs. Under elevated cutting temperatures, cutting fluid within the micro-grooves vaporizes to form a stable vapor cushion (“steam jacket”) [27,28].

As a result, cutting fluid penetrates adjacent micro-capillaries directly in the vapor phase, bypassing Stages I and II:

(12) $$\tau_{fl}=\tau_{g}\ll \tau_{total}$$

Using representative capillary dimensions (*r* = 1.5 µm, length *l_l_* =80 µm, geometry parameters *a* = 80 µm and *h* = 1.5 µm), fluid properties (*v* = 1.02 × 10^−6^ m^2^/s, *P_r_* = 6.2, *K* = 1.8 × 10^−2^, *µ_g_* = 1.55 × 10^−5^ Pa·s), and pressure boundaries (*P*_1_ = 2.51 × 10^5^ Pa, *P*_2_ = *P*_atm_ = 1.01 × 10^5^ Pa with Δ*P* = 0.15 MPa, *T* = 473 K), the calculated liquid penetration time from Equation (9) is *τ_l_* ≈ 3.82 ms, whereas the direct vapor filling time from Equation (11) is *τ_g_* ≈ 0.16 ms. This comparison yields *τ_g_*/*τ_l_* ≈ 0.042, demonstrating that direct vapor-phase filling shortens fluid delivery time by more than an order of magnitude.

Because vapor filling time *τ_g_* is nearly an order of magnitude smaller than liquid penetration time *τ_l_*, fluid delivery into the interface micro-capillaries is greatly accelerated. Rapid fluid ingress promotes the continuous formation of a boundary lubrication film, lowering the fluid boundary layer shear strength *τ_m_*:

(13) $$\tau_{m}=\tau_{m}(\omega_{g}\cdot t_{p})=f(\frac{1}{\omega_{g}\cdot t_{p}})=\tau_{min}+\;{\left(\tau_{s}-\tau_{min}\right)}^{\left(-\kappa \cdot \omega_{g}\cdot t_{p}\right)}$$

where *ω_g_* is the vapor penetration velocity, *t_p_* is the total penetration duration, and *f* represents a mathematical function.

The total tool–chip friction force *F_f_* comprises sticking friction and sliding boundary friction. For micro-textured tools, micro-grooves with surface area *A_z_* replace direct tool–chip adhesion area *A_s_* with boundary-lubricated area *A_m_*. The total tool–chip friction force *F_f_* for micro-textured tools becomes:

(14) $$F_{f}=\tau_{s}(A_{s}-A_{z})+\tau_{m}(A_{m}+A_{z})=F_{f,con\upsilon }-(\tau_{s}-\tau_{m})A_{z}$$

Because the workpiece shear strength *τ_s_* exceeds the boundary film shear strength *τ_m_* (*τ_s_* > *τ_m_*), the total friction force *F_f_* decreases by (*τ_s_* − *τ_m_*)*A_z_*. Lower friction reduces the average shear stress ${\bar{\tau }}_{mc}({\bar{\tau }}_{mc}<{\bar{\tau }}_{c})$, which decreases the friction angle *β* = arctan(*F_f_*/*F_n_*). A smaller friction angle *β* increases the workpiece shear angle *ϕ* according to Merchant’s relation:

(15) $$\phi =\frac{\pi }{4}-\frac{\beta }{2}+\frac{\gamma_{o}}{2}$$

An increased shear angle *ϕ* reduces the shear area, cutting power consumption, and heat generation. Consequently, direct vapor-phase filling in micro-textured tools simultaneously lowers cutting forces and interfacial cutting temperatures.

## 3. Materials and Methods

### 3.1. Tool Material and Laser Surface Micro-Texturing

**Figure 5:**
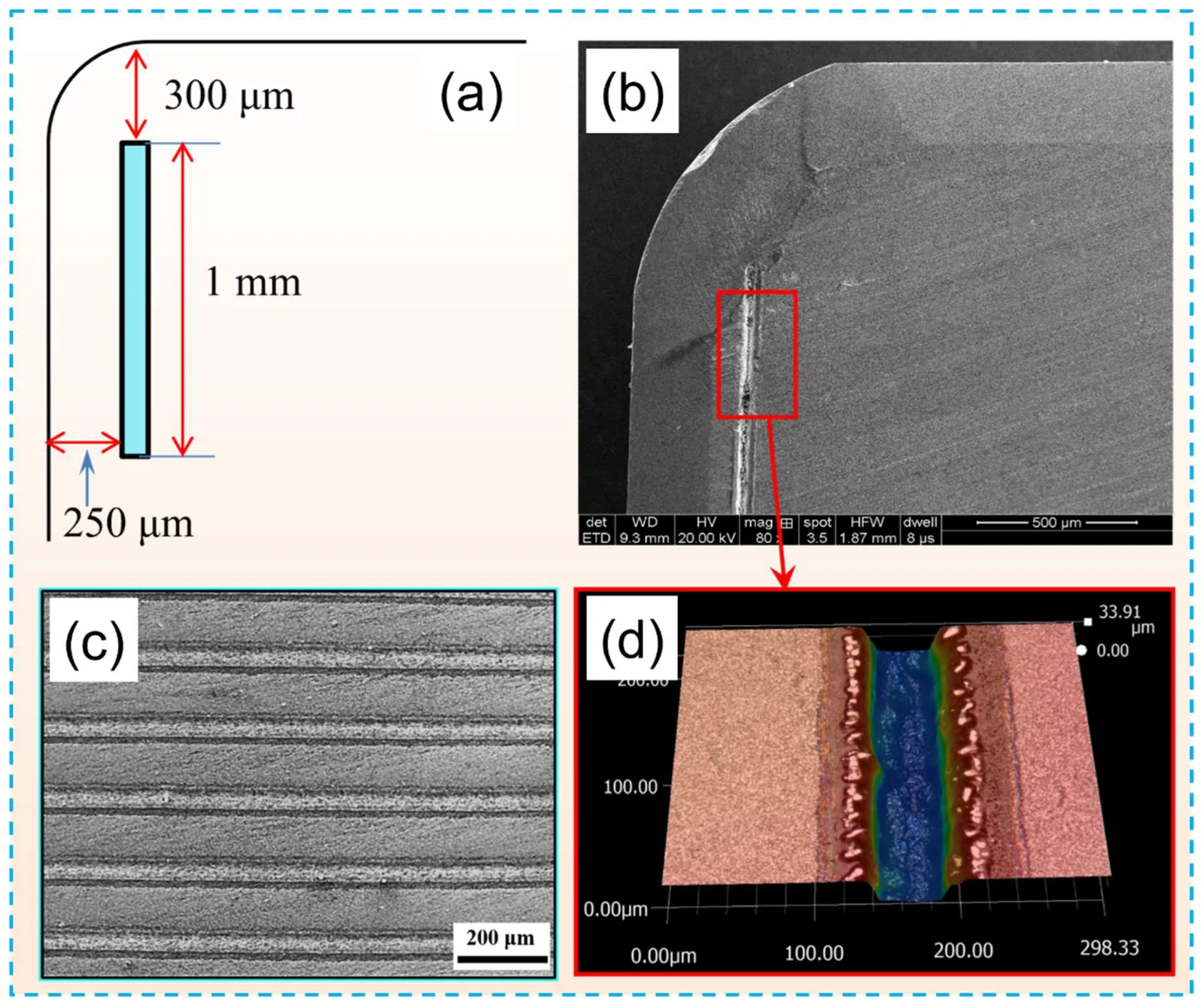
Fabrication and morphological characterization of surface micro-textured ceramic tools: (**a**) two-dimensional schematic of single-groove ceramic insert showing the distance (250 μm) behind the main cutting edge; (**b**) SEM morphology of a microtextured tool; (**c**) SEM morphology of parallel micro-grooves; (**d**) 3D optical profile showing groove width (45 μm) and depth (15 μm).

Figure 5 illustrates the two-dimensional structure, laser texturing process, and cross-sectional morphology of the surface micro-textured ceramic tools. As shown in Table 1, the Al_2_O_3_/TiC ceramic composite comprising 45 wt.% Al_2_O_3_ and 55 wt.% TiC was selected as the tool substrate material. The physical properties of the substrate include a density of 4.76 g/cm^3^, a Vickers hardness of 23.5 GPa, a flexural strength of 900 MPa, and a fracture toughness of 5.2 MPa·m^1/2^. Ceramic inserts with dimensions of 12.7 mm × 12.7 mm × 4.76 mm were ground and polished to achieve a surface roughness Ra < 0.2 μm. All specimens were cleaned ultrasonically in ethanol and acetone baths for 25 min to eliminate surface contaminants prior to laser texturing. Parallel linear micro-grooves were fabricated using a nanosecond laser processing system with a wavelength of 1064 nm, operated at an average power of 6 W, a pulse frequency of 40 kHz, a scanning speed of 150 mm/s, and a total scan count of two passes. For static fluid penetration test specimens, parallel micro-grooves were processed with a pitch of 200 μm. For cutting inserts, based on the contact mechanics described in Section 2.1, a single linear micro-groove parallel to the main cutting edge was placed at a distance of 250 μm behind the cutting edge, targeting the secondary capillary sliding zone (*l_e_*) of the rake face. The cross-sectional profile of the laser-fabricated micro-grooves was characterized using scanning electron microscopy (SEM) and a Keyence VHX-5000 3D optical super-depth-of-field microscope, revealing a uniform groove width of 45 μm and a depth of 15 μm.

### 3.2. Simulated Thermal Fluid Penetration Experiment

**Figure 6:**
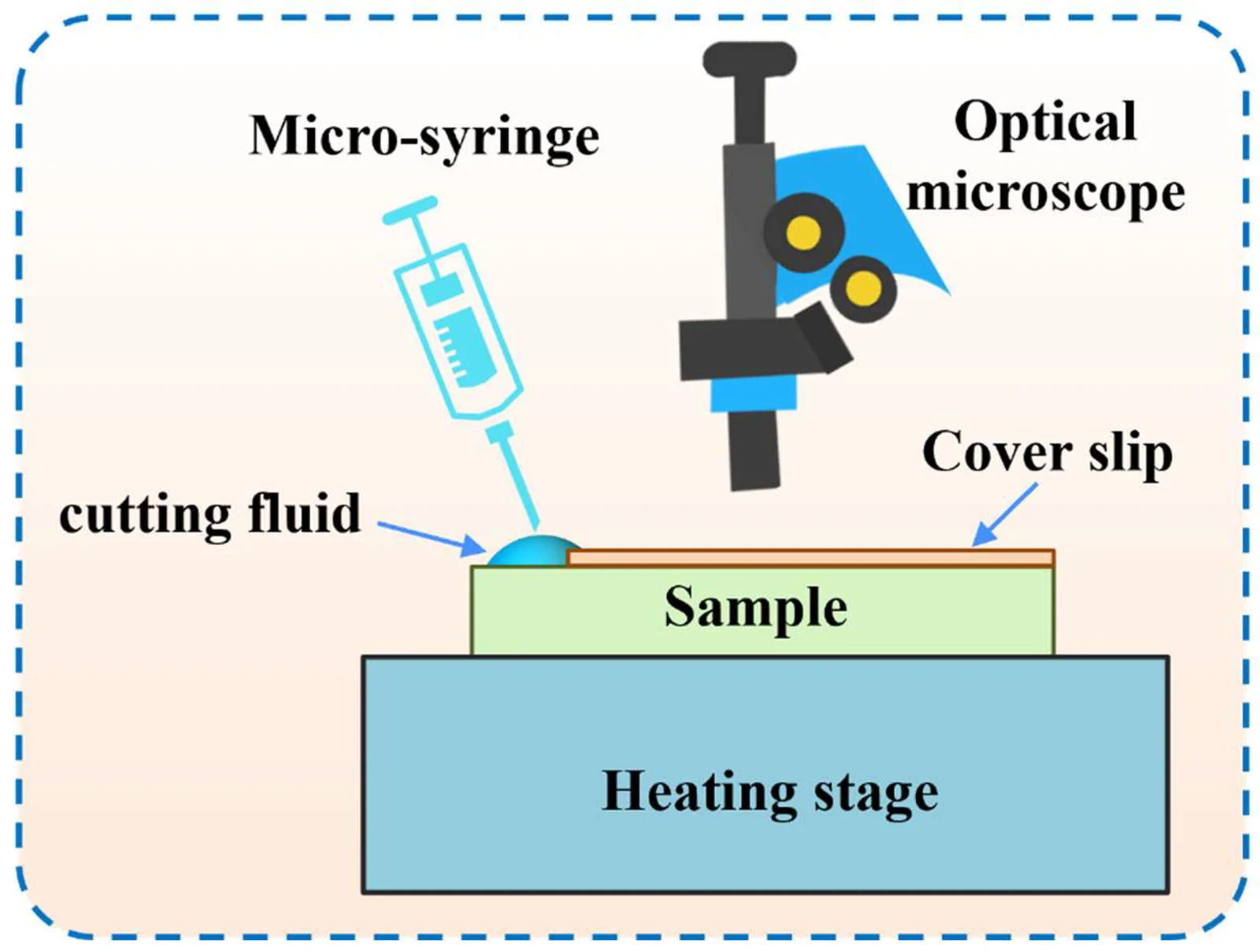
Schematic illustration of the specialized optical micro-visualization setup for simulated thermal fluid penetration tests under glass coverslip contact constraints.

Figure 6 displays the schematic of the optical micro-visualization testing platform for simulated thermal fluid penetration tests. To validate the temperature-dependent capillary phase transition model established in Section 2.3, a specialized optical visualization setup was constructed to evaluate fluid penetration and spreading kinetics under constrained contact. A glass coverslip (18 mm × 18 mm, thickness 0.15 mm) was mounted on the polished ceramic surface, establishing a constrained capillary clearance of 2.5–4.0 µm. A 5.0 µL fluid droplet was dispensed after 60 s of thermal equilibration. Phase domains were identified by optical brightness contrast and segmented via threshold binarization to calculate projected surface areas. Because the boiling point of water is about 100 °C, the liquid–gas phase transition process can be fully captured at a test temperature as high as 150 °C, serving as a conservative baseline for higher machining temperatures (200–400 °C) governed by the Clausius–Clapeyron relation.

The experiments were conducted with multiple parallel micro-grooves (pitch 200 µm) to observe macroscopic fluid spreading and multi-channel vapor–liquid distribution. Ceramic specimens with untextured (smooth) and micro-textured surfaces were mounted on a precision heating stage, with temperatures controlled between 30 °C and 150 °C at intervals of 30 °C (30 °C, 60 °C, 90 °C, 120 °C, and 150 °C). A clean glass cover slip was placed directly over the specimen surface to simulate the constrained capillary contact between the tool rake face and the chip. An emulsified cutting fluid (Solcut oil-V600, DOMINO Co., Ltd., Shanghai, China) was stably delivered dropwise along one edge of the cover slip. Dynamic fluid transport, capillary ingress, and dynamic phase transitions (liquid-phase penetration versus vapor-phase filling) were recorded in real time using a VHX-5000 optical microscope (Keyence Corporation, Osaka, Japan). Steady-state liquid and vapor penetration surface areas were quantitatively measured across different thermal conditions. It should be noted that while glass coverslips differ from metallic steel chips in thermal conductivity and surface energy, the primary objective of this optical simulation is to establish a transparent, non-deformable constrained capillary gap for real-time visualization of dynamic vapor–liquid phase transition kinetics. The thermal stage provides a controlled isothermal gradient (30–150 °C), isolating phase equilibrium behaviors from complex chip plastic deformation. All experiments were repeated three times (N = 3), and the data were averaged.

### 3.3. Metal Cutting Tests and Characterization

**Figure 7:**
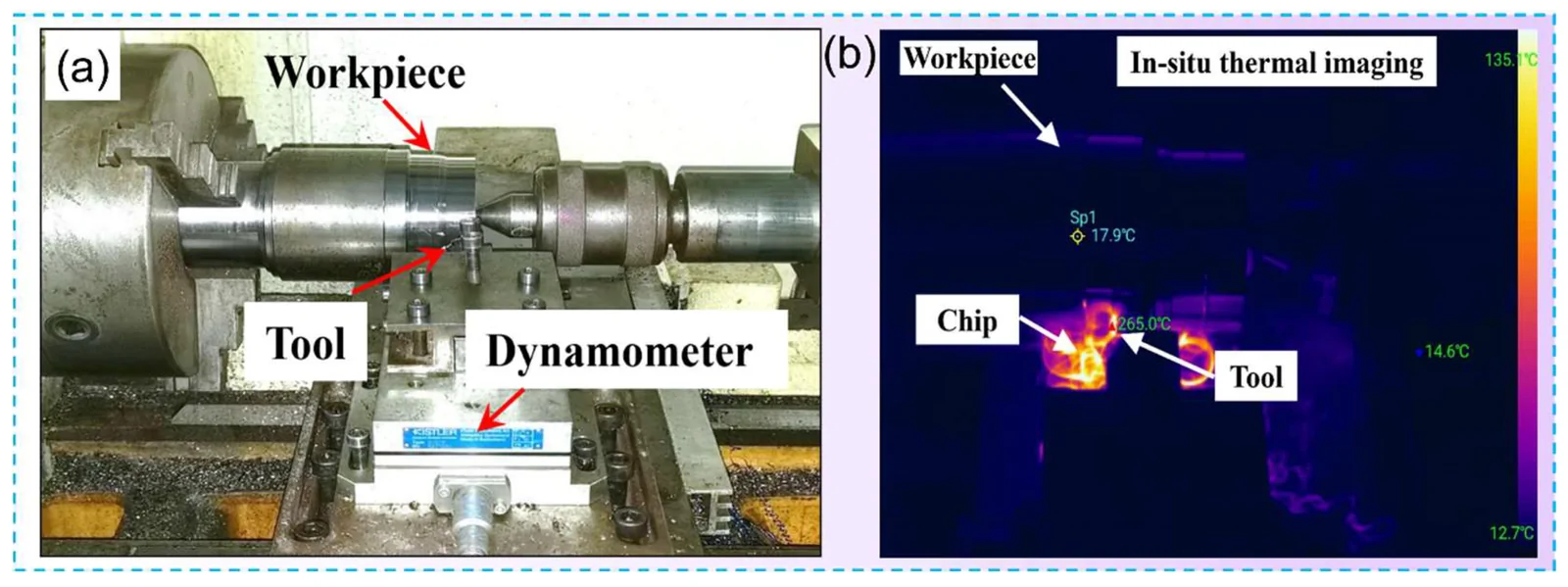
Experimental hard turning setup and instrumentation: (**a**) machining platform on CA6160 lathe equipped with a Kistler 9129AA piezoelectric dynamometer; (**b**) in situ infrared thermal imaging monitoring system.

Figure 7 shows the experimental hard turning setup on the CA6160 lathe (Shenyang Machine Tool Co., Ltd., Shenyang, China) equipped with a force dynamometer and thermal imaging instrumentation. The experiments were conducted with a single linear micro-groove placed 250 µm behind the cutting edge to preserve mechanical edge strength while targeting the sliding zone. Hard turning experiments were conducted under wet cooling conditions, using AISI 1045 hardened carbon steel (Baowu Steel Group Corp., Ltd., Shanghai, China) with a hardness of HRC 45 and an outer diameter of 110 mm as the workpiece to verify the direct vapor-filling friction reduction mechanism. Machining performance was evaluated by comparing conventional untextured ceramic tools (CTs) with single-groove micro-textured ceramic tools (TTs). The cutting geometry was set to a rake angle *γ_o_* = −5°, clearance angle *α_o_* = 5°, cutting edge inclination angle *λ_s_* = 0°, and major cutting edge angle *K_r_* = 45°. Solcut oil-V600 emulsified cutting fluid (Domino Co., Ltd., Shanghai, China) was continuously supplied to the rake face contact area at a constant flow rate of 11.2 L/min. Machining parameters were maintained at a depth of cut *a_p_* = 0.5 mm, a feed rate *f* = 0.3 mm/rev, and cutting speeds *v* ranging from 40 m/min to 240 m/min (40 m/min, 85 m/min, 124 m/min, 200 m/min and 240 m/min), with each turning test running for 3.5 min. The cutting force and temperature measurement results under various cutting conditions are summarized in Table 2. Three-component cutting forces (*F_x_*, *F_y_*, *F_z_*) were acquired in real time using a Kistler 9129AA piezoelectric quartz dynamometer (Kistler Instrumente AG, Winterthur, Switzerland). Interfacial cutting temperatures were monitored continuously using an infrared thermal camera. Cutting temperatures were acquired using an infrared camera (FLIR A655sc, Teledyne FLIR LLC, Wilsonville, OR, USA, 50 Hz, spatial resolution 25 µm/pixel, measurement uncertainty ± 2.0%) calibrated to an insert emissivity of *ε* = 0.91 ± 0.02. Because the intimate contact interface is partially obscured by the moving chip, thermal data were sampled from the exposed rake face immediately adjacent to the chip separation frontier (0.1–0.3 mm from the cutting edge), representing the apparent near-interface cutting zone temperature. Post-machining rake face morphology, micro-capillary structures, and wear features were analyzed using SEM (Quanta 250 FEG, FEI Company, Hillsboro, OR, USA) and 3D optical microscopy (VHX-5000, Keyence Corporation, Osaka, Japan). Chemical element mapping and local micro-area compositional analyses inside interface micro-capillaries were performed via X-ray energy dispersive spectrometry (EDS) to trace fluid-derived element residue (Na) and quantify workpiece material adhesion (Fe transfer) [29]. A two-sample Student’s *t*-test with a significance level of *α* = 0.05 was conducted to assess the statistical differences in cutting forces and temperatures between CTs and TTs.

## 4. Results and Discussion

### 4.1. Temperature-Dependent Fluid Penetration Dynamics

**Figure 8:**
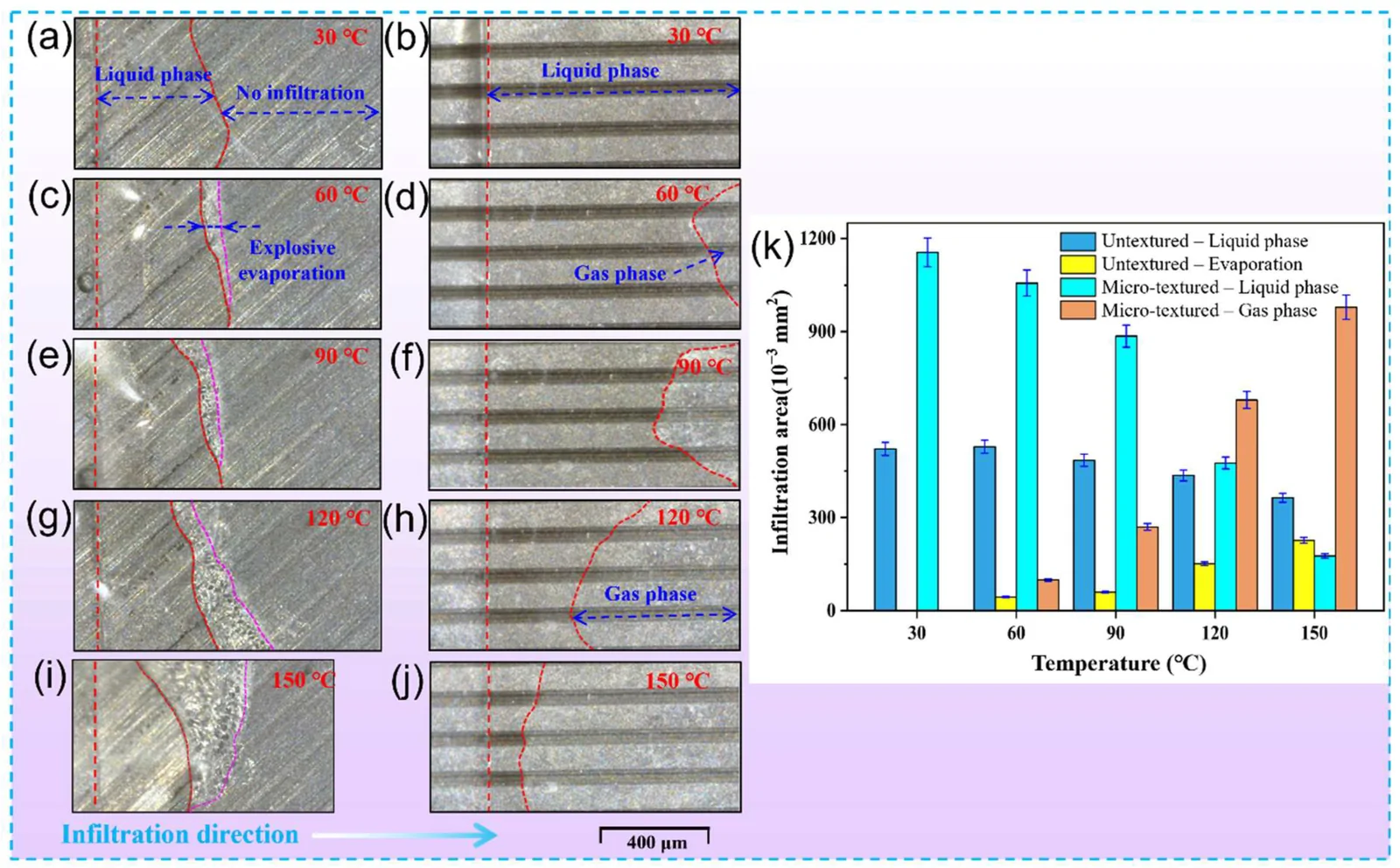
Temperature-dependent fluid penetration dynamics and phase distribution on ceramic surfaces: (**a**–**j**) optical micro-observations comparing fluid spreading on untextured (30–150 °C) and micro-textured surfaces (30–150 °C); (**k**) quantitative comparison of liquid, evaporation, and vapor penetration areas across temperatures.

Figure 8 compares the temperature-dependent fluid penetration dynamics and phase distributions on untextured and micro-textured ceramic surfaces. On the untextured (smooth) surface, cutting fluid penetrates exclusively in the liquid phase at 30 °C. As the temperature increases from 60 °C to 150 °C, fluid evaporation and micro-explosions occur along the liquid border. The liquid penetration area on the untextured surface decreases by 31.1% at 150 °C, while the evaporation area reaches its maximum value of 226.6 × 10^−3^ mm^2^. In contrast, the micro-textured surface establishes a dynamic equilibrium between liquid and vapor phases. As temperature rises, the liquid phase area contracts by 83.3%, whereas the vapor phase area expands dramatically from 103.2 × 10^−3^ mm^2^ at 60 °C to 978.5 × 10^−3^ mm^2^ at 150 °C. The vapor area on the micro-textured surface at 150 °C is over four times larger than the evaporation area on the smooth surface.

These phenomena can be explained by the physical mechanisms derived in Section 2.3 and Section 2.4. On smooth surfaces, fluid transport follows the conventional three-stage ingress path. High thermal energy causes localized boiling near the contact edge, creating an evaporation barrier that restricts deep liquid penetration. On micro-textured surfaces, micro-grooves act as localized heat-exchanging reservoirs. High temperatures rapidly vaporize the stored fluid, forming a continuous vapor cushion (“steam jacket”). This vapor cushion supplies gaseous lubricant directly into adjacent contact micro-capillaries. This observation is physically consistent with the direct vapor-phase filling mechanism modeled in Equation (12). Unlike the sequential droplet evaporation model proposed by Godlevski et al. [2], micro-textures enable fluid to bypass the time-consuming liquid ingress stage (*τ_l_*). The elevated temperature accelerates vapor kinetic energy, expanding the effective gaseous lubrication coverage.

### 4.2. Interfacial Micro-Capillary Ingress and Chemical Trace Analysis

**Figure 9:**
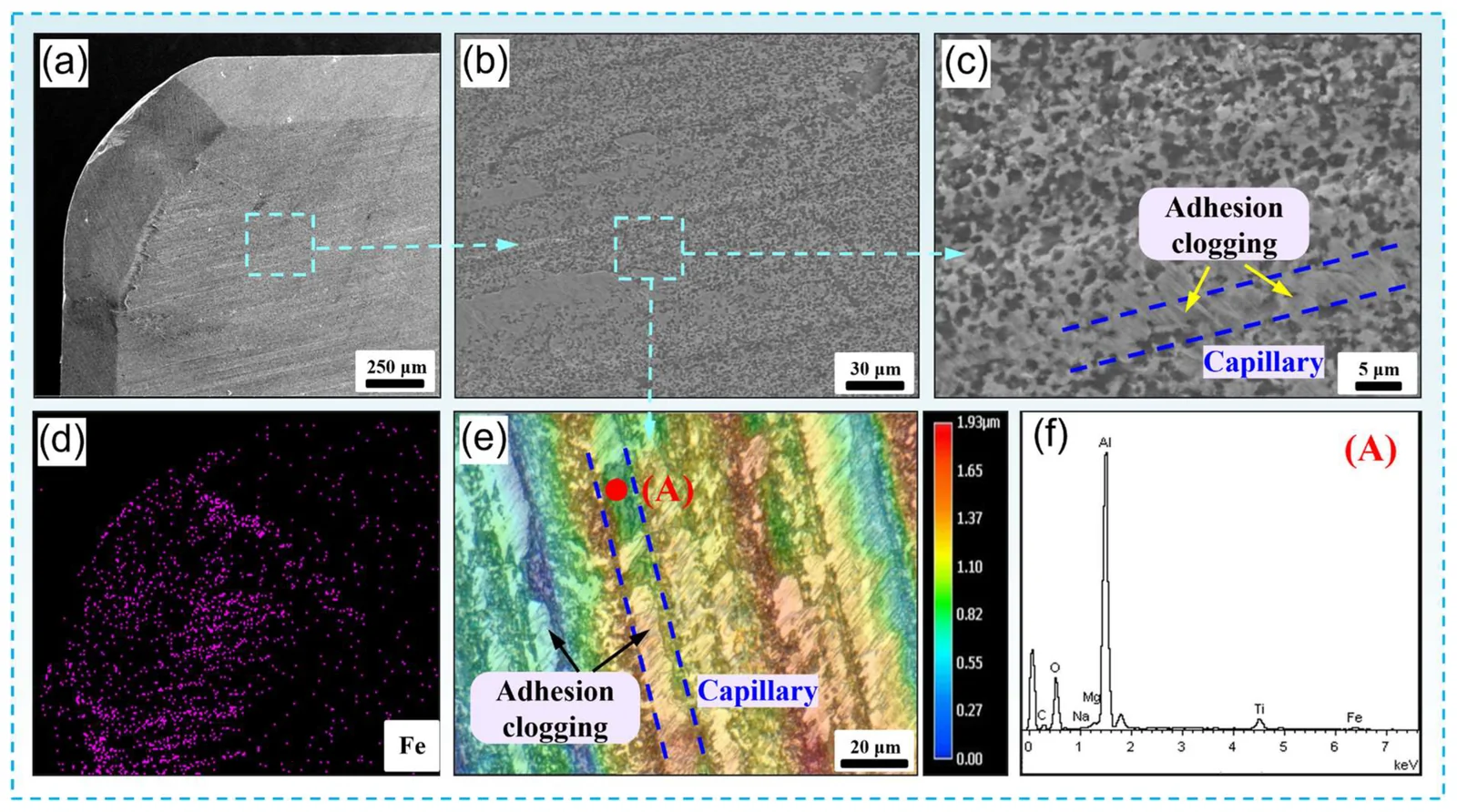
Rake face wear morphology and chemical element analysis of conventional ceramic tool CT after hard turning (*v* = 240 m/min, *t* = 3.5 min): (**a**) SEM panorama of worn rake face; (**b**,**c**) magnified SEM images showing severe capillary clogging by transferred material; (**d**) Fe element mapping distribution; (**e**) 3D optical morphology of clogged capillary; (**f**) EDS energy spectrum at Point A showing absence of fluid elements.

Figure 9 highlights the rake face wear morphology, capillary clogging, and EDS compositional spectra of the conventional tool CT after hard turning (*v* = 240 m/min). Microscopic observations of worn tool rake faces after hard turning (*v* = 240 m/min, *t* = 3.5 min) reveal distinct capillary contact features. On the conventional tool (CT), interface micro-capillaries are heavily clogged by transferred workpiece material [30]. Energy dispersive spectrometry (EDS) at Point A shows a high iron concentration (6.04 at.% Fe) alongside ceramic substrate elements (Ti, C, Al, O). However, no fluid-derived chemical elements are detected inside the CT capillaries.

**Figure 10:**
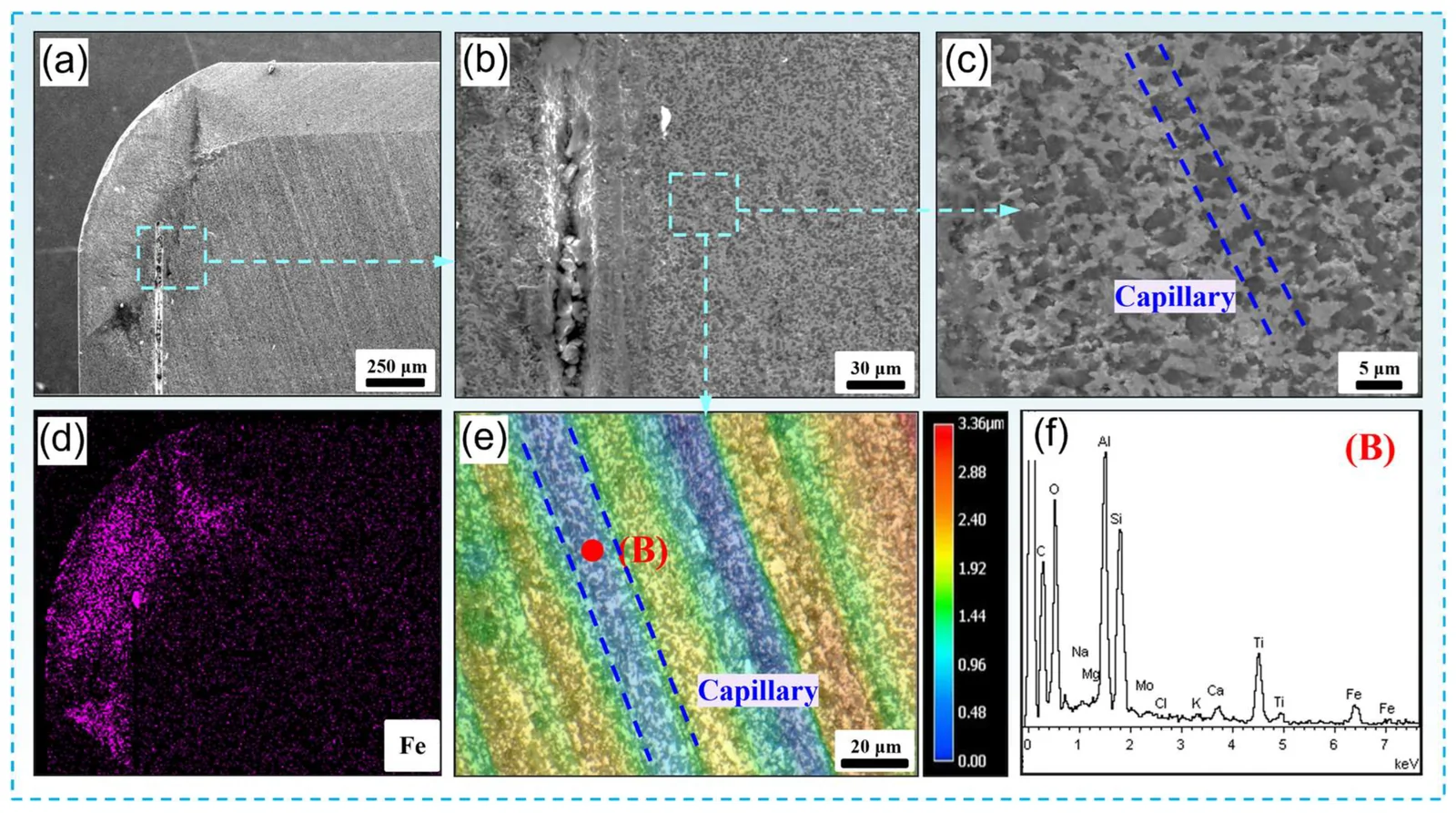
Rake face wear morphology and chemical tracer analysis of micro-textured ceramic tool TT after hard turning (*v* = 240 m/min, *t* = 3.5 min): (**a**) SEM panorama showing micro-groove location; (**b**,**c**) magnified SEM images showing open, unblocked micro-capillaries; (**d**) Fe element mapping showing adhesion suppression; (**e**) 3D optical morphology of open capillary; (**f**) EDS energy spectrum at Point B highlighting fluid-derived Na chemical tracer.

Figure 10 presents the worn surface morphology, open capillaries, and fluid-derived Na chemical tracer inside the micro-textured tool (TT). On the micro-textured tool (TT), interface micro-capillaries remain unblocked and clear of severe metal transfer. EDS analysis at Point B inside the TT capillaries detects 0.98 at.% sodium (Na), 0.11 at.% magnesium (Mg), and trace amounts of chlorine (Cl) and calcium (Ca). Concurrently, the iron content drops drastically from 6.04 at.% to 0.11 at.%.

The presence of sodium (Na) inside the TT capillaries provides chemical evidence of cutting fluid ingress into the tool–chip interface. Commercial Solcut oil-V600 emulsion contains sodium petroleum sulfonate (R-SO_3_Na) as its primary water-soluble surfactant and corrosion inhibitor. Because sodium is completely absent from both the Al_2_O_3_/TiC ceramic substrate and the AISI 1045 steel workpiece, Na serves as a reliable chemical tracer originating exclusively from the fluid additives [31]. Wide-area EDS mapping (Figure 9d and Figure 10d) corroborates the local point analyses, demonstrating broad suppression of Fe adhesion across the textured contact zone. This result indicates that cutting fluid successfully enters the secondary sliding capillaries (*l_e_*) via the micro-groove supply channels. In conventional tools, extreme compressive normal stresses (*σ*_0_) seal the capillary openings. The fluid cannot penetrate before plasticized workpiece material fills and clogs the capillary voids. In micro-textured tools, the micro-grooves lower localized normal stress and supply vaporized fluid under pressure. The gaseous fluid penetrates the capillaries rapidly, forming a protective boundary film. This boundary film lowers interfacial shear strength and suppresses workpiece material adhesion. This observation offers deeper chemical confirmation of fluid permeation within the interface capillaries.

Rake face examinations after the 3.5 min turning tests (Figure 9 and Figure 10) show that tools remain in the initial running-in wear stage. On the conventional tool (CT), material transfer dominates the contact zone, causing adhered layer build-up rather than fully developed crater cavity gouging. On micro-textured tool (TT), the pre-fabricated micro-groove (depth 15 µm) retains its open geometry without severe degradation, while the tool land shows negligible crater wear depth (*K_T_*), confirming that boundary vapor lubrication protects the rake face during steady-state machining.

### 4.3. Machining Tribological Performance: Cutting Forces, Temperature, and Friction

**Figure 11:**
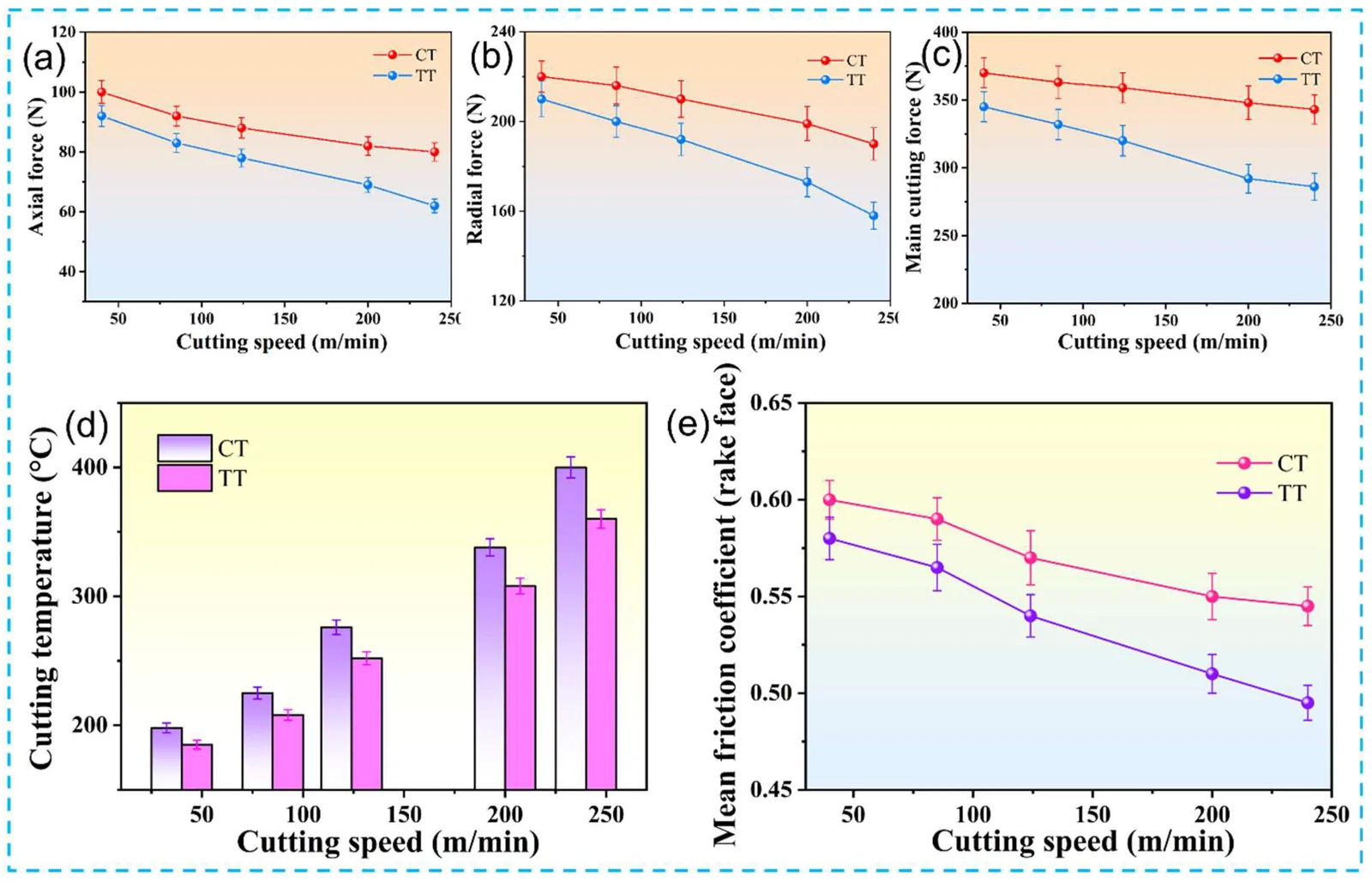
Tribological performance comparison between conventional (CT) and micro-textured (TT) ceramic tools under various cutting speeds (*v* = 40–240 m/min): (**a**) axial force *F_x_*; (**b**) radial force *F_y_*; (**c**) main cutting force *F_z_*; (**d**) interfacial average cutting temperature; (**e**) tool–chip average friction coefficient *μ* = *F_f_*/*F_n_*.

Figure 11 summarizes the tribological performance comparison between conventional tool (CT) and micro-textured tool (TT) across various cutting speeds. All cutting trials and force/temperature acquisitions were repeated three times (N = 3), with data reported as mean ± standard deviation. Two-sample Student’s *t*-tests confirm that micro-textured tool (TT) produced statistically significant reductions in main cutting force *F_z_* (*p* < 0.05) across all speeds: 370 ± 6.2 N vs. 345 ± 5.5 N at 40 m/min (*p* = 0.018) and 343 ± 5.1 N vs. 286 ± 4.8 N at 240 m/min (*p* = 0.004). Cutting temperatures also exhibited statistically significant drops from 400 ± 8.5 °C (CT) to 360 ± 7.2 °C (TT) at 240 m/min (*p* = 0.009). Machining test results demonstrate significant performance improvements for micro-textured tools across all tested cutting speeds (40 m/min−240 m/min). Both tools show a declining trend in axial force *F_x_*, radial force *F_y_*, and main cutting force *F_z_* as cutting speed increases. This general reduction is caused by thermal softening of the workpiece at higher cutting speeds [32]. Compared to CT, tool TT reduces axial forces *F_x_* by 8–22%, radial forces *F_y_* by 5–17%, and main cutting forces *F_z_* by 7–17%. Force reductions become increasingly pronounced at higher cutting speeds.

Average cutting temperatures increase monotonically with cutting speed for both tools. However, TT consistently yields lower cutting temperatures than CT. The temperature reduction percentage increases with cutting speed, showing drops of 6.5%, 7.5%, 8.7%, 8.9%, and 10.0% at 40 m/min, 85 m/min, 124 m/min, 200 m/min, and 240 m/min, respectively. Furthermore, the tool–chip friction coefficient *μ* decreases with cutting speed. Tool TT exhibits a lower friction coefficient than CTs at all speeds. The reduction in *μ* expands from 3.3% at 40 m/min to 9.2% at 240 m/min (dropping from 0.545 to 0.495).

The tribological improvements can be explained by the analytical model in Section 2.4. As shown in Equation (14), micro-grooves replace high-shear adhesion contact (*A_s_*) with low-shear boundary lubrication contact (*A_m_*). Rapid vapor-phase penetration lowers the boundary shear strength (*τ_m_* < *τ_s_*), directly reducing the total friction force *F_f_*. Lower friction force decreases the friction angle *β*, which, in turn, increases the shear angle *ϕ* according to Equation (15). A larger shear angle reduces the primary shear area, lowering plastic deformation work and main cutting force *F_z_* [32]. Less plastic deformation work and lower friction work reduce total heat generation rate (*q_r_*), resulting in lower cutting temperatures. At higher cutting speeds, higher interfacial temperatures accelerate fluid vaporization within the micro-grooves. Faster vaporization increases vapor penetration velocity (*ω_g_*), reducing penetration time (*τ_fl_*) and enhancing boundary film replenishment. Consequently, the friction reduction and cooling effects of micro-textured tools become more significant under high-speed cutting conditions.

### 4.4. Academic Significance, Comparative Insights, and Limitations

This study provides critical physical insights into green machining and tool surface engineering. Previous studies on textured tools often attributed tribological benefits solely to reduced contact area or mechanical debris trapping [18,33]. Previous studies have demonstrated force reductions using micro-grooved ceramic tools, but have focused primarily on dry cutting conditions [19]. Under wet conditions, researchers often observed inconsistent lubrication due to fluid penetration barriers. By identifying the direct vapor-phase filling mechanism, this work provides critical physical insights into how cutting fluid reaches extreme pressure contact zones. The findings indicate that surface micro-textures act not merely as lubricant reservoirs, but also facilitate localized phase transitions that transform liquid coolant into high-velocity gaseous lubricant.

Despite these contributions, certain limitations should be acknowledged. First, the current cutting experiments evaluated a single linear micro-groove geometry placed 250 µm from the cutting edge. The effects of groove density, cross-sectional shape, and multi-array patterns require systematic optimization. Second, the analytical model assumes steady-state vapor transport, whereas actual chip sliding involves high-frequency dynamic pressure fluctuations. Third, testing was conducted over a 3.5 min duration. While this study focuses on steady-state force, temperature, and penetration kinetics over a 3.5 min cutting regime, the progressive evolution of crater wear depth (*K_T_*) and long-term micro-groove durability under extended industrial production cycles remain important topics for future investigation. Additionally, the EDS analysis was conducted at representative local points, and comprehensive multi-region statistical sampling warrants future investigation.

Placing the micro-groove 250 μm behind the edge targets the sliding/separation zone (*l_e_*), where natural chip up-curl prevents severe plastic groove filling during machining of hardened AISI 1045 steel. For highly ductile materials such as titanium alloys or austenitic stainless steels, severe lateral plastic flow may cause groove entrapment if placed too close to the cutting edge (<150 μm), where narrower grooves (<25 μm) or solid-lubricant infilling are more effective. Preserving an untextured 250 μm land protects ceramic edge strength and suppresses progressive crater wear (*K_T_*), thereby promoting long-term tool life.

## 5. Conclusions

This study investigated the fluid penetration kinetics and friction reduction mechanisms of micro-textured Al_2_O_3_/TiC ceramic tools. The main conclusions are drawn as follows:

(1) Surface micro-textures facilitate cutting fluid transport into interface micro-capillaries via a vapor-phase pathway, bypassing the conventional multi-stage liquid ingress process. This mechanism reduces fluid penetration time into capillaries by nearly an order of magnitude and significantly lowers boundary shear stress. The experimental observations and tribological improvements are consistent with the proposed direct vapor-phase filling mechanism.
(2) Micro-grooves maintain dynamic vapor–liquid equilibrium under high thermal gradients (30–150 °C). At 150 °C, the vapor transport area on micro-textured surfaces reaches 978.5 × 10^−3^ mm^2^, over four times larger than the evaporation zone on smooth surfaces.
(3) EDS analysis detects fluid-derived sodium (Na, 0.98 at.%) inside micro-textured capillaries, supporting fluid ingress into secondary sliding contact zones. Consequently, workpiece material adhesion (Fe transfer) inside micro-capillaries drops drastically from 6.04 at.% to 0.11 at.%.
(4) Micro-textured tools can reduce the main cutting force by up to 17%, cutting temperature by up to 10.0%, and reduce the tool–chip friction coefficient by 9.2% at a cutting speed of 240 m/min. Higher cutting speeds accelerate fluid vaporization kinetics, further enhancing boundary lubrication.

## Figures and Tables

**Table 1:** Physical and mechanical properties of Al_2_O_3_/TiC ceramic cutting inserts.

Composition (wt.%)	Density (g/cm^3^)	Vickers Hardness (GPa)	Flexural Strength (MPa)	Fracture Toughness (MPa·m^1/2^)	Thermal Conductivity (W/m·K)	Thermal Expansion Coefficient (10^−6^/K)
Al_2_O_3_/55%TiC	4.76	23.5	900	5.2	28	7.8

**Table 2:** Summary of cutting parameters, tool geometry, and cooling conditions.

Category	Parameter	Value/Specification
Tool Geometry	Rake angle *γ_o_*/Clearance angle *α_o_*	−5°/5°
	Inclination angle *λ_s_*/Cutting edge angle *K_r_*	−0°/45°
	Groove distance from cutting edge	250 µm
	Groove width × depth	45 µm × 15 µm
Cutting Conditions	Workpiece material	AISI 1045 hardened carbon steel (HRC 45)
	Cutting speed *v*	40, 85, 124, 200, 240 m/min
	Depth of cut *a_p_*/Feed rate *f*	0.5 mm/0.3mm/rev
	Cutting time per trial	3.5 min
	Number of replications	3 trials per condition
Lubrication	Fluid type/Flow rate	Solcut oil-V600/11.2 L/min

## Data Availability

The original contributions presented in this study are included in the article. Further inquiries can be directed to the corresponding author.
